# Cardiac Troponin T and Illness Severity in the Very-Low-Birth-Weight Infant

**DOI:** 10.1155/2012/479242

**Published:** 2012-02-22

**Authors:** Danielle N. Lopes, José M. Moraes Ramos, Maria Elizabeth Lopes Moreira, Jofre A. Cabral, Manoel de Carvalho, José Maria de Andrade Lopes

**Affiliations:** ^1^Neonatal Intensive Care Unit, Clínica Perinatal Laranjeiras, 22240-002 Rio de Janeiro, Brazil; ^2^Department of Neonatology, Instituto Fernandes Figueira, Oswaldo Cruz Foundation, 22250-020 Rio de Janeiro, Brazil

## Abstract

*Introduction*. Respiratory distress are very common in Very-low-birth-weight (VLBW) infants and Myocardial injury may play a role in the disease outcome. Cardiac troponin T (cTnT) is the most useful marker of injury in adult population, but has not been extensively studied in this population. *Aim*. To study the role of cTnT in VLBW infants and its association with clinical outcomes. *Methods*. All VLBW infants admitted to our NICU were included in the study. Echocardiography and blood samples for cTnT determination were collected at 24 and 48 hours of life, and values >0.1 ng/mL were considered CTnT-positive values. *Results*. A total of 116 neonates had their blood samples collected. The median cTnT concentration within 24 hours was 0.191 (0.1–0.79) ng/mL and within 48 hours was 0.293 (0.1–1.0) ng/mL. A logistic regression analysis showed that PDA, low GA, and use of dopamine were independently associated with positive cTnT and abnormal Dopplerfluxometry and diuretics use had protective effects and was independently associated with troponin values. *Conclusion*. We observed a high prevalence of positivecTnT values in VLBW infants associated with illness severity. Our findings suggest that cTnT may be a useful and early marker of myocardial injury in VLBW infants.

## 1. Introduction

Significant therapeutic and technological progress have occurred in the intensive neonatal care in the last decades, but the care of very-low-birth-weight (VLBW) newborns still remains a big challenge. These premature newborns are very often in critical conditions, are immature, and represent more than 50% of neonatal deaths [1]. 

RDS affects close to 50% of the VLBW infants; signs and symptoms of respiratory and cardiovascular disease are very similar [2], and decreased myocardial function and low cardiac output are common complications of conditions such as respiratory distress syndrome and perinatal asphyxia [3].

 The cardiac troponin T (cTnT) is the best biochemical marker for myocardial ischemia in adults. Its blood level increases within two hours after a myocardial infarct and may remain elevated for two weeks [4]. The initial studies conducted in children and newborns used less sensitive assays—of first and second generation, and the results of the cTnT were frequently negative. Most studies that used the third-generation assays seem more promising and have shown high levels of cTnT in infants with asphyxia, respiratory distress, and hypotension although there are still very few data for very-low-birth-weight infant [5, 6].

We designed this prospective study to assess the role of cTnT in the first 48 hours of life and its association with clinical outcomes in a population of very-low-birth-weight infants.

## 2. Methods

We prospectively studied all infants with birth weight less than 1500 g, admitted to the Neonatal Intensive Care Unit of Laranjeiras Perinatal Clinic, between January 2004 and July 2005. Infants with congenital malformation or genetic syndromes confirmed by laboratorial and clinic tests were excluded from the study.

 Blood samples (0.3 mL) were collected with 24 and 48 hours of life in heparinized syringes, taken to the laboratory to be stored for further analysis. The biochemical analysis was conducted via the Immunoassay Elecsys Troponin T STAT (Roche). We arbitrarily defined a positive test if cTnT blood levels were >0.1 ng/dL.

Echocardiograms were performed in the first 72 hours of life by a cardiologist consultant. The parameters recorded by the echocardiogram were mild, moderate, or severe dysfunction, cardiac dilatation, and persistence of ductus arteriosus.

Clinical data of prenatal care, multiparity, hypertension, diabetes, steroid use, fetal distress, prolonged rupture of membranes (more than 24 hours), perinatal infection, and type of delivery were recorded. Neonatal variables included Apgar scores, birth weight, gestational age, CRIB, and SNAPPE-II, as well as need for resuscitation in the delivery room.

We followed infants up to discharge and registered most important neonatal morbidities: RDS, pneumonia, use of surfactant, need of oxygen, length of mechanical ventilation, duration of nasal CPAP, hypotension and persistence of ductus arteriosus, use of indomethacin, use of inotropic drugs (dopamine, dobutamine) and diuretics, the presence of infection at admission, and antibiotic use. For statistical analysis we used the *t*-test, Non-parametric tests, chi-square and logistic regression.

The study was approved by the Ethics Committee Review Board of the Institution.

## 3. Results

Between January 1st, 2004, and July 31st, 2005, 156 pre-term infants with less than 1500 g were admitted to the high-risk nursery of Laranjeiras Perinatal Clinic. We excluded 37 infants due to death before 24 hours of life and congenital malformations. Additional 3 infants were excluded because we were unable to obtain troponin values. Therefore, the prospective study was conducted in 116 patients within this period.

The main maternal morbidities were arterial hypertension (41.2%), multiple gestations (24.4%), and the prolonged rupture of membranes (17.6%). Infection related to premature birth was found in 12.7% of the patients. The use of pre-natal steroids was a common practice (76.3%), and maternal Dopple rfluxometry was abnormal in 56 patients (47.5%).

Mean Apgar scores at 1 and 5 minutes were 6.2 ± 2.1 and 8.3 ± 1.1, respectively. A total of 95 newborns (80.5%) required resuscitation in the delivery room. The ventilation with positive pressure (VPP) was the most used procedure, followed by orotracheal intubation (36.4%). Seventy-eight preterm infants (65.5%) presented RDS, and surfactant was used in 58% of cases. Nasal constant positive airway pressure (CPAP) was used in 73.5% of VLBW infants, for 11.3 ± 10.6 days and median of 8 days. A total of 95 infants required oxygen therapy with a median of 12 days of therapy.

Sixty infants (55.5%) had a diagnosis of PDA; among those, 45.4% were treated with indomethacin and 10.1% had the ductus surgically ligated.

A total of 86 patients had an echocardiogram done in the first week of life. Severe dysfunction was found in 5.9%, moderate dysfunction in 5%, and mild dysfunction in 13.5%; and 35.3% presented cardiac overload. Pulmonary hypertension was diagnosed in 9.2% of patients.

 The mean hospital stay was 62.7 ± 36.1 days with median of 58 days.

Table 1 shows characteristics of the studied population.

One hundred and sixteen samples were obtained within 24 hours (70 samples with values ≤0.10 or undetectable). At 48 hours, we obtained 102 samples (57 samples with values ≤0.10 or undetectable). For the positive samples (values >0.10), the median cTnT at 24-hour was 0.193 (0.1–0.79 ng/mL) and at 48 hours it was 0.293 (0.1–1.0 ng/mL).

We used the 24-hour results of cTnT in the comparisons below. Several factors were associated to the increase of cTnT in the univariate analysis. Tables 2 and 3 illustrate the main categorical and continuous variables studied.

### 3.1. Multivariate Analysis: Logistic Regression

We conducted a logistic regression analysis and included in the model all variables included in the univariate analysis and considered the cardiac troponin T as dependent variable. Among the regression models tested, we chose the one that seemed more adequate, both from the statistical and biological standpoint. A patent ductus arteriousus and dopamine use increased the chance of a newborn presenting positive cTnT in 3.6 (0.95–13.3) and 5.68 (1.5–20.8) times, respectively. On the other hand, the presence of abnormal Doppler and diuretic use reduced the chance of a newborn presenting positive cTnT in 0.27 (0.09–0.7) and 0.12 (0.02–0.5), respectively. The increase in the gestational age of the newborn has a protective effect with an odds ratio of 0.65 (0.5–0.8).

## 4. Discussion

Cardiac troponin T and I (cTnT, cTnI) are accepted as the gold standard assay for diagnosis of myocardial infarction in adults. In the last decade a large number of publications confirmed the clinical usefulness of troponin T in patients with acute coronary syndrome establishing the diagnosis of ischemia, myocardial necrosis, and the prognosis of acute myocardial infarct. Due to the absolute specificity of cTnT regarding the myocardial tissue and its high sensitivity, even in microscopic regions, for myocardial necrosis, the American College of Cardiology and the European Society of Cardiology recommended cardiac troponins as the best biological markers for myocardial infarct [4].

There are several studies published on the behavior of cTnT in newborns, in different clinical situations, and its value in diagnosing cardiovascular dysfunction. Adamcová et al. studying the cTnT in the blood cord of newborns suggested that its increase would indicate fetal distress and myocardial compromise. These authors evaluated cord blood cTnT in 15 healthy term newborns and found plasma concentrations of 0.05 ± 0.04 ng/mL in 10 of the 15 infants studied. Among these, 5 showed higher concentrations of cTnT (0.19 ± 0.07 ng/mL) [5].

 In 2000, Trevisanuto et al. described high cTnT concentrations in preterm infants with respiratory distress and suggested that it was due to myocardial damage. They also observed a significant relationship of troponin T levels with the use of vasopressors, mechanical ventilation, and high oxygenation index in patients with respiratory distress syndrome. The mean plasma troponin was 0.38 ng/mL in patients with respiratory distress versus 0.13 ng/mL in patients without respiratory distress (*P* < 0.01) [2].

These same authors, in a subsequent study, compared the cTnT, cTnl, and CKMB concentrations in the blood cord of 85 healthy full-term newborns and in blood samples of their mothers' right after birth. The concentrations of cTnT and cTnl were higher than the detection limit in 2 (2.3%) and 41 (48.2%), respectively. Two mothers (2.3%) had cTnT levels higher than the detection limit; none showed increase in the cTnl levels. These authors concluded that cTnT in the blood cord is often above the detection limit at birth, while the cTnl is more often undetectable and comparable to that of healthy mothers. Therefore, these cardiac regulatory proteins are of neonatal origin and are not influenced by maternal levels [6].

Several publications have attempted to correlate increased cTnT with other markers of myocardial dysfunction and echocardiographic abnormalities. Cruz et al. studied 27 ELBW infants and measured cTnt in the first 48 hours of life. They observed increased levels in infants with low Apgar scores and requiring inotropic support. cTnT did not correlate with simultaneous echocardiographic measures of cardiac function [7]. Costa et al. And Rajakumar et al. in two separate studies found a correlation between increased cTnT and signs of myocardial damage in infants with perinatal asphyxia [8, 9].

More recently El-Khuffash et al. developed a predictive model using echocardiographic data, cTnT, B-type natruretic peptide, and a PDA scoring system in 60 infants with less than 32 weeks and less than 1500 g, who were followed until two years of age. They concluded that the use of biochemical markers and the PDA scoring system may provide a better approach to the management of PDA in the first 48 hours of life, with improved intact long term survival [10].

Our study was conducted with the aim of characterizing the behavior of the cTnT in the very-low-birth-weight newborn. We conducted prospective measurements of cTnT using third-generation assay method, for all newborns admitted to a neonatal NICU, for eighteen months, in a series of nonselected patients and observed increased values in 40% of the infants, according to our definition.

We recorded clinical events and perinatal morbidity in this group, looking for associations with the cTnT levels. Measurements were made between 24 and 48 hours of life, based on adult data after a myocardial injury. The value of 0.1 ng/mL for a cutoff was chosen according to the studies conducted in adults by Alpert et al., Ohman et al., and Costa et al. in newborns [4, 7, 8]. 

We found an association of increased cTnT values with low birth weight and gestational age and a positive association with several variables related to illness severity (Table 3).

Similar to our study, Clark et al. studied a total of 162 newborns (113 healthy and 49 sick) and observed more elevated values of cTnT in sick preterm. A multiple regression analysis demonstrated that the rise of cTnT was associated to the need of respiratory support and to the use of inotropic drugs [3].

Szymankiewicz et al. studied 39 asphyxiated newborn versus 44 nonasphyxiated newborns and tried to relate the cTnT to echocardiographic findings of myocardial damage. The cTnT was measured within 12 and 24 hours of life. The asphyxiated infants had higher levels of cTnT (0.141 versus 0.087 ng/mL) than the nonasphyxiated infants (*P* < 0.01) [11].

In our study, two variables related to asphyxia showed a positive association with cTnT: the need for intubation in the delivery room and the mean Apgar scores of 1st and 5th minute. Our findings are similar to many other studies that suggested a positive association of cTnT and asphyxia in the neonatal period [2, 3, 8–10].

We performed a logistic regression analysis adjusting for neonatal morbidities and severity scores. The best model showed that an abnormal maternal doppler and the use of diuretics had a protective effect, while low gestational age, the persistence of ductus arteriosus, and the use of dopamine were associated with a positive cTnT.

The frequency of maternal arterial hypertension and changes in the Doppler fluxometry was high in this population (42% and 47%). These are the most common maternal risk factors for elective interruption of pregnancy. Abnormal Doppler fluxometry, when conjugated with intrauterine growth retardation, is associated to adverse neonatal outcome. However, in the absence of growth retardation, premature delivery may render better fetus and so a protective effect.

Previous publications have already related increased cTnT to a patent ductus arteriousus. El-Khuffash and Molloy studied 80 infants with 28-week median gestational age and observed increased cTnT levels in infants with an open ductus which decreased after ductal closure [12].

Important hemodynamic changes may be related to an open ductus arteriousus. There is frequently atrial and ventricular overload and a drop of diastolic blood pressure causing so-called blood flow steel. The blood that flows in the descending aorta during systole comes back through the patent channel towards the pulmonary artery. Large shunts may lead to more than 50% return of blood in the descending aorta, causing hypoperfusion of all systemic arteries, including the coronary arteries. It is possible that the coronary hypoperfusion will lead to myocardial ischemia, some degree of necrosis and increased cTnT values. Improvement in cardiac overload with a patent ductus may also explain the protective effect of diuretics [13].

The effect of gestational age is still a controversial issue. Different from our findings, previous studies did not show increased cTnT values infants with lower gestational age [3, 10, 12, 14]. Most studies had smaller numbers of VLBW infants, and very few babies below 28 of weeks gestation. In a more recent series from our institution, not published yet, including only babies with less than 1000 g, we have also not found a relationship between cTnT values and gestational age suggesting that these differences may be population related [15].

Our study is the largest series of very-low-birth-weight newborns studied, with a third-generation cTnT assay. The predominant profile associated with a positive troponin test, was that of an immature baby, intubated in the delivery room, with respiratory distress, and requiring vasoactive drugs.

Our findings confirm previous observations and suggest that cTnT may be a very useful and early marker of myocardial injury in very-low-birth-weight newborn infants.

## Figures and Tables

**Table 1:** Main Characteristics of the Studied Population (*n* = 116).

Birth weight (g)	1050 ± 306
Gestational age (weeks)	29.2 ± 2.3
CRIB	4.1 ± 3.9
SNAPPE	22.2 ± 2.4
Mechanical ventilation	61.9%
Persistence of Ductus Arteriosus	55.5%
Use of Dopamine	34.5%
Use of Dobutamine	37%
Early sepsis	23.5%
Deaths	6.7%

VPP = Ventilation with positive pressure.

**Table 2:** Neonatal variables versus cardiac troponin T results.

Variables	Troponin (−) *n* = 70	Troponin (+) *n* = 46	*P* value
Weight (g)	1124 ± 292	953 ± 301	*P* < 0.003
GA (weeks)	30 ± 2.0	28 ± 2.2	*P* < 0.01
CRIB (Me)	3.1 ± 3.4	5.5 ± 4.3	*P* < 0.001
SNAPPE (Me)	18.5 ± 16.4	27 ± 17.4	*P* < 0.01
Apgar 1 min (Me)	6.7 ± 1.9	5.3 ± 2.2	*P* < 0.01
Apgar 5 min (Me)	8.5 ± 1.0	7.9 ± 1.3	*P* < 0.01
Days on MV	3.91 ± 9.1	12.5 ± 20.1	*P* < 0.01
Days on CPAP	5.3 ± 7.9	12.9 ± 12.2	*P* < 0.001
Total Time of O_2_	16.1 ± 25.4	37.3 ± 43.1	*P* < 0.002
Days in hospital	54.2 ± 27.9	75.14 ± 43.7	*P* < 0.006

**Table 3:** Neonatal variables versus cardiac troponin T results.

Variables	Troponin (−)	Troponin (+)	*P* value
Prenatal steroids	74%	77%	*P* < 0.9
Abnormal Doppler	54%	37%	*P* < 0.12
EOT (delivery room)	25%	53%	*P* < 0.005
DMH	55%	80%	*P* < 0.01
Use of surfactant	45%	76%	*P* < 0.002
Hypotension	24%	34%	*P* < 0.30
PDA	42%	74%	*P* < 0.01
Use of indomethacin	34%	60%	*P* < 0.01
Use of dopamine	24%	52%	*P* < 0.004
Use of dobutamine	27%	54%	*P* < 0.005
Use of diuretics	42%	58%	*P* < 0.13
Cardiac dysfunction	15%	40%	*P* < 0.03
Cardiac overload	28%	47%	*P* < 0.05
